# Treatment of pediatric patients and young adults with particle therapy at the Heidelberg Ion Therapy Center (HIT): establishment of workflow and initial clinical data

**DOI:** 10.1186/1748-717X-7-170

**Published:** 2012-10-17

**Authors:** Stephanie E Combs, Kerstin A Kessel, Klaus Herfarth, Alexandra Jensen, Susanne Oertel, Claudia Blattmann, Swantje Ecker, Angelika Hoess, Eike Martin, Olaf Witt, Oliver Jäkel, Andreas E Kulozik, Jürgen Debus

**Affiliations:** 1 Department of Radiation Oncology, University Hospital of Heidelberg, Im Neuenheimer Feld 400, Heidelberg, 69120, Germany; 2 Department of Pediatric Hematology and Oncology, University Hospital of Heidelberg, Im Neuenheimer Feld 430, Heidelberg, 69120, Germany; 3 Heidelberg Ion Therapy Center (HIT), Im Neuenheimer Feld 450, Heidelberg, 69120, Germany; 4 Department of Anaesthesiology, University Hospital of Heidelberg, Im Neuenheimer Feld 400, Heidelberg, 69120, Germany; 5 CCU Pediatric Oncology, German Cancer Research Center, Im Neuenheimer Feld 280, Heidelberg, 69120, Germany

**Keywords:** Proton radiation, Carbon ion radiotherapy, Children

## Abstract

**Background:**

To report on establishment of workflow and clinical results of particle therapy at the Heidelberg Ion Therapy Center.

**Materials and methods:**

We treated 36 pediatric patients (aged 21 or younger) with particle therapy at HIT. Median age was 12 years (range 2-21 years), five patients (14%) were younger than 5 years of age. Indications included pilocytic astrocytoma, parameningeal and orbital rhabdomyosarcoma, skull base and cervical chordoma, osteosarcoma and adenoid-cystic carcinoma (ACC), as well as one patient with an angiofibroma of the nasopharynx. For the treatment of small children, an anesthesia unit at HIT was established in cooperation with the Department of Anesthesiology.

**Results:**

Treatment concepts depended on tumor type, staging, age of the patient, as well as availability of specific study protocols. In all patients, particle radiotherapy was well tolerated and no interruptions due to toxicity had to be undertaken. During follow-up, only mild toxicites were observed. Only one patient died of tumor progression: Carbon ion radiotherapy was performed as an individual treatment approach in a child with a skull base recurrence of the previously irradiated rhabdomyosarcoma. Besides this patient, tumor recurrence was observed in two additional patients.

**Conclusion:**

Clinical protocols have been generated to evaluate the real potential of particle therapy, also with respect to carbon ions in distinct pediatric patient populations. The strong cooperation between the pediatric department and the department of radiation oncology enable an interdisciplinary treatment and stream-lined workflow and acceptance of the treatment for the patients and their parents.

## Introduction

In pediatric oncology, most treatment concepts in the 21^st^ century are of curative intent, aiming at long-term overall survival. With continuous increase in tumor control and cure, also side effects of the treatments, especially long-term side effects, have moved even more into focus. With respect to radiotherapy, sparing of normal tissue and reduction of integral dose is the most effective means to prevent treatment-related side effects. Over the last decades, advances in radiation oncology from conventional 2D- to 3D-radiation therapy, to the establishment of high-precision stereotactic radiotherapy and subsequently intensity-modulated radiotherapy (IMRT) have continuously enabled the radiation oncologist to deliver higher local doses even to complex target volumes while sparing surrounding organs at risk (OAR). However, intermediate and low doses of radiation remain to be applied. One way of reducing integral dose and potentially reducing side effects is the use of particle therapy, due to the physical properties of ion beams [1,2]).

The distinct physical characteristics of a particle beams potentially offer clinical benefit especially in pediatric oncology: In the entry channel of the beam almost no energy is deposited, while a high local dose deposition, the Bragg Peak, can be guided by energy-variation of the beam, into the defined target volume. Dose distributions are characterized by a steep dose-fall-off at the field borders, and the integral dose can be reduced significantly. Therefore, proton radiotherapy has a central role in pediatric oncology, and should be applied when available. In clinically active proton centers such as the Paul-Scherrer-Institute (PSI) in Villigen, Switzerland, at Francis H. Burr proton center in Boston, or at MD Anderson Cancer Center in Houston, proton radiotherapy has been established in clinical routine for paediatric patients. Clinical results in different tumor entities such as rhabdomyosarcoma, ependymoma or medulloblastoma have shown very low toxicity and promising outcome [3-12]. Moreover, comparative analyses with photon treatment have shown that reduction of integral dose may lead to a significant decrease in side effects, especially with respect to secondary malignancies in pediatric patients treated with proton radiotherapy [13]. However, until now no real long-term data, especially no randomized comparisons of high-end photons and protons are available.

Carbon ions, additionally to the physical benefits of particle beams, offer a higher relative biological effectiveness (RBE) which can be exploited also in pediatric oncology for very radioresistant tumors, such as osteosarcomsas, difficult to treat with low-LET radiotherapy [14].

To effectively compare particle therapy with advanced photon techniques, multiple field treatment planning as well as active beam application is required. Modern particle therapy centers are equipped with active beam delivery, as well as with gantries to allow for variable beam positions. At the Heidelberg Ion Therapy (HIT) center, active beam delivery using the rasterscanning technique is available for particle therapy. Moreover, the center is equipped with two horizontal treatment rooms, as well as an ion gantry for carbon and proton treatments [14].

Since November 2009, HIT is treating patients within clinical routine offering carbon and proton radiotherapy for adults and pediatric patients alike. The close collaborations with the Department of Pediatric Hematology and Oncology and the Department of Anesthesiology enable a strong interdisciplinary treatment of children as in- or outpatients.

In the present work we report our initial experience of particle therapy in pediatric patients with and without anesthesia focusing on the establishment of workflow, interaction between departments as well as initial clinical results.

## Patients and methods

### Patient selection

From January 2009 to November 2011, we treated 36 pediatric patients (aged 21 or younger) with particle therapy at HIT. All patients were seen and the treatment concepts were discussed in an interdisciplinary team. This project was performed in accordance with institutional ethical policies. All data was collected from our institutional patient database and tumor registry. They were collected anonymously using a uniform retrieval code for data acquisition.

Median age was 12 years (range 2-21 years), five patients (14%) were younger than 5 years of age. Indications included pilocytic astrocytoma, parameningeal and orbital rhabdomyosarcoma, skull base and cervical chordoma, osteosarcoma and adenoid-cystic carcinoma (ACC), as well as one patient with an angiofibroma of the nasopharynx. In all but one patients radiotherapy was performed as primary radiation treatment. Only one patient with a recurrent rhabdomyosarcoma (RMS) of the skull base was treated as re-irradiation. Patients’ characteristics are summarized in Table 1.

**Table 1:** Patients’ characteristics of 36 children and young adults treated with protons and carbon ions at the Heidelberg Ion Therapy Center (HIT)

***Histology***	***N (%)***
*primary brain tumors*	*10 (28%)*
pilocytic astrocytoma	n = 7
glioblastoma	n = 1
ATRT	n = 1
PNET	n = 1
chordoma/chondrosarcoma	10 (28%)
skull base chordoma	n = 7
skull base chondrosarcoma	n = 3
*osteosarcoma*	*3 (8%)*
Skull base	n = 2
pelvic	n = 1
*rhabdomyosarcoma*	*4 (11%)*
*ACC*	*3 (8%)*
*other sarcoma*	*4 (11%)*
*angiofibroma*	*1 (3%)*
**Gender**	
male	11 (31%)
female	25 (69%)
***Radiotherapy with anesthesia***	
yes	4 (11%)
no	32 (89%)
**Treatment concept**	
Protons	16 (44%)
Carbon ions	13 (36%)
Photon + Carbon Ion Boost	6 (+ 1 Proton Boost) (20%)

### Treatment planning

Particle therapy was applied using the intensity modulated rasterscanning technique with the horizontal beamline. Sixteen patients were treated with protons (44%), and 13 patients (36%) were treated with carbon ions (C12). C12 was mostly applied for young adults with skull base tumors, and protons were mostly used for pediatric patients. In 7 patients (20%) with difficult-to control tumors (glioblastoma, osteosarcoma and adenoid-cystic-carcinoma (ACC)) requiring high local doses, particle therapy as a boost treatment to the macroscopic tumor was combined with photon IMRT.

Treatment planning as well as radiation therapy was performed in individual fixation devices manufactured individually for each patient. For brain and skull base tumors, either Scotch Cast™ or thermoplastic mask systems were used allowing an overall repositioning accuracy of 1-2 mm. For thermoplastic mask systems, patient positioning was conducted with the laser system in the treatment room using Beekley-Spots^®^ on the mask or with three target points tattooed and marked with metal fiducials during the planning CT scan. For Scotch Cast™ Cast masks, patient setup was performed using stereotactic setup. Pelvic tumors were immobilized using individual vacuum bags or a combination of knee and foot rests. Target volume definition was based on CT- and MR-Imaging; amino-acid PET or FDG-PET was used for treatment planning depending on the tumor type and the respective clinical situation. Depending on the tumor entity and tumor site as well as the postoperative situation, we defined target volumes according to ICRU criteria including a gross tumor volume (GTV), clinical target volume (CTV) as well as a planning target volume (PTV).

Target volume definition is conducted on every slice of the three-dimensional data cube using the Siemens *Dosimetrist* and *Oncologist* software tools (Siemens, Erlangen, Germany). For treatment planning, the system “Syngo PT Planning” developed by Siemens Oncology Care Systems (OCS, Erlangen, Germany) was used, offering all modern aspects of 3D treatment planning, which was specifically developed for planning of scanned proton and ion beams. It includes three-dimensional treatment planning based on biological plan optimization using the approach of the Local Effect Model (LEM). The LEM is a generic model allowing for RBE-calculation in different tissue types and for selected endpoints, which was validated in various preclinical calculations and by the treatment of over 450 patients at the Gesellschaft für Schwerionenforschung (GSI) [15]. The optimization of the scan control parameters for the raster scanning technique within the treatment planning system (TPS) is done with respect to the biological effective dose of the particles. A fixed value for the relative biological effectiveness (RBE) of 1.1 is used clinically for proton treatments. For carbon ions, the optimization is based on the LEM model with tumor- and normal-tissue specific α/β values.

#### Patient positioning

To validate patient positioning prior to each fraction of particle therapy using an orthogonal X-ray imaging system, correlation of planning CT DRRs with the orthogonal X-ray focusing mainly on bony landmarks was used for position corrections. This registration process and the subsequent performance of the calculated correction vector were supervised by a radiation oncologist together with the radiation therapist. Position correction was performed using re-positioning of the treatment couch as well as using the pitch-and-roll feature of the robotic table system in some patients.

### Anesthesia

**Figure 1:**
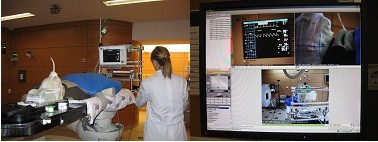
**Clinical workflow for treatment under anesthesia in cooperation with the Department of Anesthesiology (A).** A mobile anesthesia device was connected to the observation console of the radiation therapy treatment room; continuous monitoring of the patient was possible during patient setup and irradiation from outside the treatment room (**B**).

For the treatment of small children, an anesthesia unit at HIT was established in cooperation with the Department of Anesthesiology. For this purpose, a dedicated anesthesia room was built equipped with the required instruments and medication. A transportable anesthesia system (Draeger, Lübeck, Germany) was installed on the treatment table in the particle therapy treatment room (Figure 1). A risk analysis has been performed according to the medical device directive. For observation of vital parameters, relevant information is transmitted into the treatment control rooms via realtime datalines for continuous monitoring of the small children. A specialized team of pediatric anaesthesiologists performs anesthesia for each treatment fraction. Application of anesthesia is performed in the treatment room, and dedicated data lines ensure secure observation of vital parameters during irradiation.

### Follow-up visits

All patients were seen for regular follow-up visits initially 4-6 weeks after completion of radiotherapy, thereafter in 2-3 months intervals or as required clinically. Patients were seen by the Department of Pediatric Hematology and Oncology as well as the Radiation Oncology Department. No patient was lost to follow-up.

Tumor control was documented with imaging on each follow-up visit, depending on the tumor type. Additional examinations including endocrinological follow-up were scheduled as required.

Treatment-related side effects were documented according to the Common Terminology Criteria for Adverse Events (CTCAE) Version 4.1.

## Results

### Treatment concepts

Treatment concepts depended on tumor type, staging, age of the patient, as well as availability of specific study protocols. In general, pediatric patients are treated within study protocols of the German Society for Pediatric Hematology and Oncology, and target volumes as well as dose prescription were performed accordingly. These were dependent on histology and location, e.g. for low-grade gliomas, a dose of 50.4 Gy - 54 Gy E was applied in 1.8 Gy fractions using protons, and for the orbital rhabdomyosarcoma a median total dose of 52.2 Gy in single fractions of 1.8 Gy was applied using protons.

For ACC, which is rarely seen in pediatric patients and young adults, the treatment concept was based on the established regimen for adult patients, including photon radiotherapy and a carbon ion boost to the primary tumor region after incomplete resection [16]. In general photon radiotherapy is given to the areas of microscopic spread up to 50 Gy, and the carbon boost is applied up to 18 Gy E; in a most recent trial, the boost escalation to 24 Gy E was evaluated, the trial has finished recruitment, and early data showed overall tolerability [17]. Specific protocols at the Heidelberg Center have been established for certain difficult to treat tumor entities, including osteosarcoma and glioblastoma [18]. For glioblastoma, the CLEOPATRA-protocol evaluates a carbon ion boost to the macroscopic tumor identified by amino-acid-PET and T1-weighted contrast-enhancing MRI [19]. Photon radiotherapy is applied to the resection cavitiy and T2-Hyperintense region with a total dose of 50 Gy E in 2 Gy E fractions, and the carbon ion boost in the experimental arm is applied up to 18 Gy E in 3 Gy E fractions. In the control arm, proton radiotherapy is applied up to the standard dose of 60 Gy E in 5 fractions à 2 Gy E after the photon part.

For patients with osteosarcoma, a carbon ion boost to the macroscopic tumor after proton radiotherapy is evaluated within a single-armed phase II trial [20]. Chemotherapy is added according to the most recent multimodality protocols in this indication (COSS/EURAMOS). For chordoma and chondrosarcoma patients, target volume definition is comparable to treatment concepts in the adult population [21,22].

#### Tolerability of treatment

In all patients, particle treatment was well tolerated and no interruptions due to toxicity had to be undertaken; planned radiation series could be completed applying the prescribed doses. Toleration of positioning devices worked well in most patients, for smaller children without anesthesia training sessions to tolerate mask fixations were held. Four patients aged 4 years and younger were treated with anesthesia. One patient suffered from a local atypical rhabdoidtumor (ATRT) of the brain stem, one patient was diagnosed with an infratentorial pilocytic astrocytoma, one with a large optic glioma, and one Ewing’s sarcoma of the right maxillofacial region.

The median follow-up time was 9 months (range 1-23 months). No patient was lost to follow-up.

**Figure 2:**
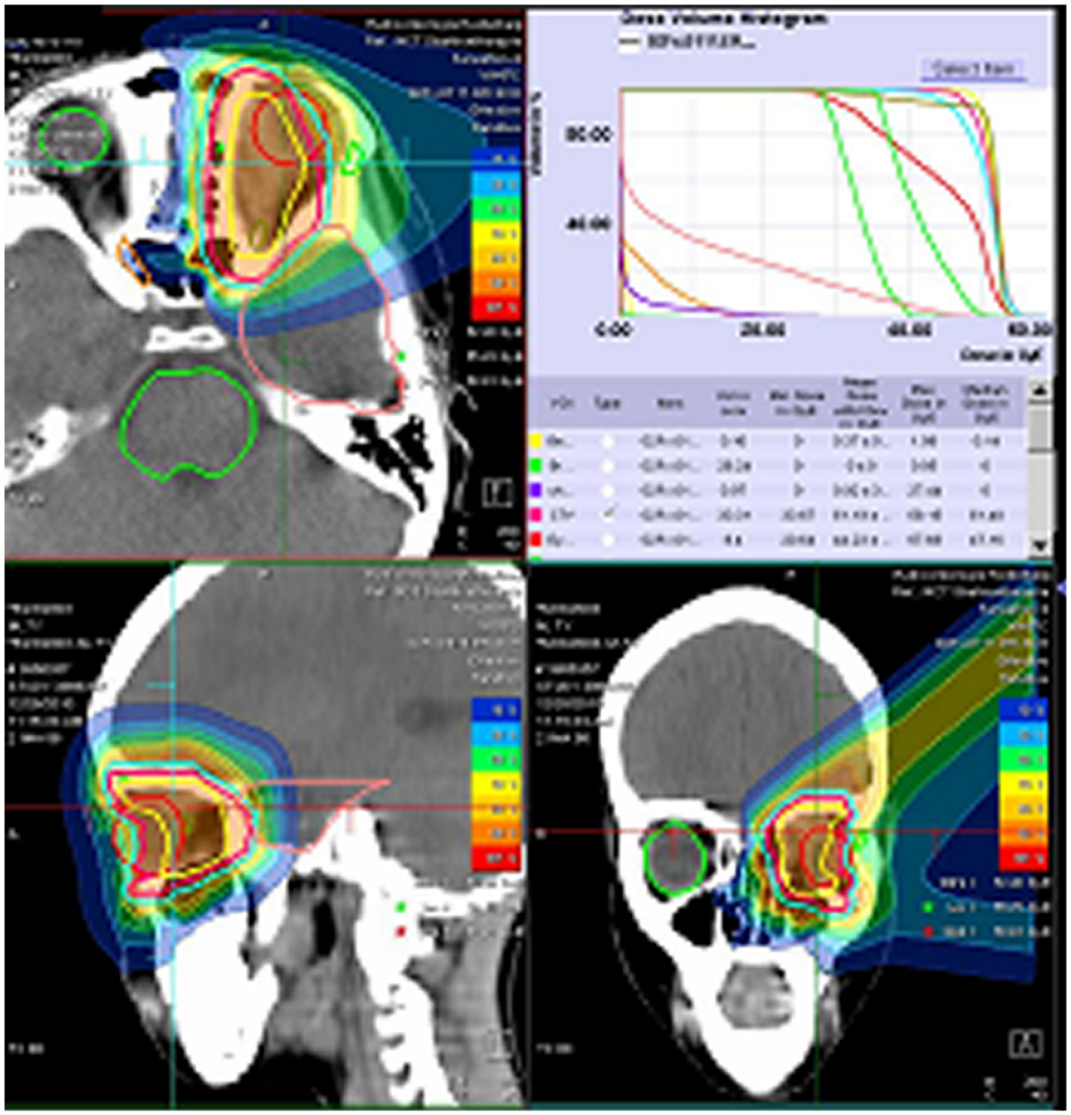
Two-field proton treatment of a 5 years-old child with an orbital rhabdomyosarcoma.

During follow-up, only mild toxicites were observed including hair loss in patients with superficial lesions. Two patients with base-of-skull tumors developed middle-ear effusion towards the end of treatment. Six patients developed mucositis CTCAE Grade 1-2; patients suffered from large base-of-skull tumors and ACCs, as well as one patient with an oropharyngeal synovial sarcoma and one large rhabdomyosarcoma. No severe acute effects were observed. Specific region-correlating toxicities included one patient with an orbital rhabdomyosarcoma treated with protons showing a mild conjunctivitis to the end of treatment. The treatment plan is shown in Figure 2.

A 16-years-old male patient treated for a rhabdomyosarcoma of the skull base with positive cervical lymph nodes was treated with hyperfractionated-accelerated proton radiotherapy according to the soft tissue sarcoma study protocol (CWS) up to a total dose of 51.2 Gy E in single doses of 1.6 Gy E, twice daily. The patient developed mucositis CTC Grad II-III with difficulty swallowing during treatment, which resolved completely until the first follow-up visit. However, this patient developed an early recurrence with distant meningeal spread along the craniospinal axis, however, the local primary tumor region remains stable.

During follow-up, hyperpigmentation in the RT portals were observed in two patients, one with an orbital rhabdomysarcoma, and one with a sarcoma of the larynx.

### Treatment response and local control

During follow-up, only one patient died of tumor progression: Carbon ion radiotherapy was performed as an individual treatment approach in a child with a skull base recurrence of the previously irradiated rhabdomyosarcoma. Local tumor recurrence was observed at first follow-up 4 weeks after the end of treatment, and palliative care was decided on an interdisciplinary consensus.

Besides this patient, tumor recurrence was observed in two additional patients: A 18 years old female with a glioblastoma treated with a carbon ion boost developed local tumor recurrence 13 months after primary diagnosis. As salvage treatment, chemotherapy was intensified. Another 16 years old boy treated for a rhabdomyosarcoma of the skull base with positive cervical lymph nodes developed progression as meningeal spread along the craniospinal axis with tumor cells in the craniospinal fluid (CSF) 6 months after proton treatment. Salvage treatment consisted of chemotherapy, as well as irradiation of the craniospinal axis avoiding the previously irradiated regions with a total dose of 36 Gy, and a boost to 50 Gy to the visible macroscopic lesions, with helical tomotherapy.

All other patients remained stable during follow-up with no signs of clinical or imaging defined tumor progression. Tumor response was observed in several patients depending on histology, most predominantly in ACC, sarcoma and primary brain tumor patients. Clinical response was also observed in patients with skull base lesions including resolution of occulomotor and abducens pareses in 2 patients. Pain in the sacral region improved significantly after carbon ion radiotherapy for a sacral osterosarcoma, and hemipareses improved correlating with imaging-response in a glioblastoma patient.

**Figure 3:**
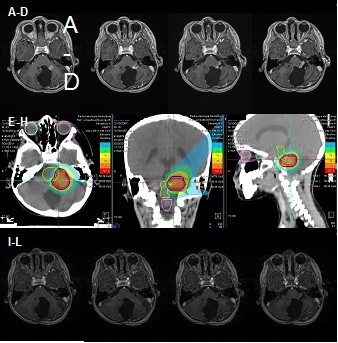
**Treatment of a patient with an infratentorial pilocitic astrocytoma.** Imaging for Treatment planning (**A-D**), treatment plan for protons, 2 fields, total dose of 54 Gy E in single doses of 1.8 Gy E (**E-H**), and imaging response 3 months after treatment (**I-L**).

**Figure 4:**
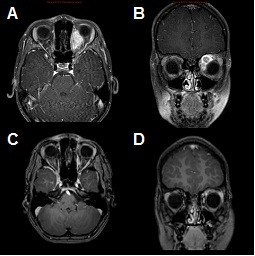
**Imaging response of a 5 years-old child with an orbital rhabdomyosarcoma (plan shown Figure**4**).** Inital imaging for treatment planning (**A;B**), Follow-up imaging 6 months after treatment; imaging showed a significant reduction in contrast enhancement with an overall reduction of the lesion in diameter.

A four-year old child with an infratentorial pilocytic astrocytoma was treated for tumor progression with anesthesia, during follow-up, the lesion showed a significant reduction in size, as well as a clear response with respect to contrast-enhancement which almost resolved completely (Figure 3). The patient treated for an orbital rhabdomyosarcoma with severe exophthalmia prior to treatment demonstrated significant reduction in tumor volume during follow-up. Clinically, exophthalmia resolved almost completely, and only a slight hyperpigmentation in the area of the treatment portals remained during follow-up (Figure 4).

## Discussion

Particle therapy for the curative treatment of children and young adults requires special demands on all ranks: Indication for treatment must be decided in an interdisciplinary setting, keeping in mind several important factors such as patient age, tumor type, staging and risk group, existing treatment concepts and running study protocols, as well as the special care necessary for small children requiring anesthesia for treatment. A strong focus must be set on target volume definition, delineation of organs at risk (OAR), choice of the optimal beam angles and dose distribution aiming at a reduction of dose to normal tissue. The reduction of integral dose is a main concern in pediatric radiation oncology, and the physical properties of ion beams offer a benefit compared with even advanced photon techniques due to the low dose deposition in the entry channel of the beam, and the high local dose deposition within the Bragg peak. Clinical evaluation and long-term data are currently being generated within several clinical protocols.

At HIT, treatment of pediatric patients with different indications was established since the beginning of patient treatment in 2009. Since then, 36 patients with different tumor entities were treated with proton and carbon ion radiotherapy. A streamlined clinical workflow for even very young children in anesthesia was established by a close collaboration with a dedicated team of anaesthesiologists for pediatric anesthesia. The strong collaboration with the neighboring departments of neurosurgery and pediatric oncology enable efficient and patient-oriented medical care. In all patients, few toxicities were observed, which were only mild and related to the size and anatomical lesion of the patients. Especially, frequent side effects such as hair loss in patients treated for brain tumors can be reduced significantly, depending on the location of the lesion. No severe acute or long-term side effects could be documented. Imaging response was observed in a number of patients, and all patients despite three remained stable during follow-up. Recurrences occurred in three patients, one child treated for a recurrence of a skull base rhabdomyosarcoma, and another patient developed meningeal spread along the craniospinal axis based on a cervical rhabdomyosarcoma, and in one patients with a glioblastoma. The safety and efficacy of proton and carbon ion treatments delivered by active rasterscanning could therefore be demonstrated.

The potential benefit of particle therapy for pediatric patients is the possibility to reduce integral dose, and thus reduce the risk for radiation-induced side effects, such as secondary malignancies. Is has been shown that taking into account several risk estimates, particle treatments compare favorably to photon treatments, especially when using beam scanning compared to beam scattering [23,24]. Several proton centers have reported excellent clinical outcome in various tumor entities:

At MGH in Boston, the treatment of patients with parameningeal rhabdomyosarcoma was studied based on elaborate treatment planning comparisons, evaluating the optimal treatment available [24]; In a group of 17 children treated with protons with a median age of 3.4 years (range, 0.4-17.6), a median dose of 50.4 Gy E was applied [3]. After a median follow-up time of 5 years, 59% remained failure-free, and overall survival was 64%. Late effects related to proton radiotherapy in the 10 recurrence-free patients included reduction in overall height in three patients, endocrinopathies (n = 2), mild facial hypoplasia (n = 7), failure of permanent tooth eruption (n = 3), dental caries (n = 5), and chronic nasal/sinus congestion in two patients. For Ewing’s sarcoma, as well, only mild side effects were observed in a group of 30 children, with tumors originating from various anatomical regions [25]. After a median proton dose of 54 Gy E, 3-years event-free survival rate was 60%, with local control and overall survival at 86% and 89%. Side effects included mostly mild reactions of the skin, despite four hematologic malignancies often observed after topoisomerase and anthacycline chemotherapy. For primary brain tumors, such as ependymomas, proton radiotherapy has shown convincing initial clinical results in 17 patients after a median follow-up of 26 months. Local control was 86%, and overall survival was 89%, with the extent of surgery being the most prominent prognostic factor [8]. Other studies have reported similarly convincing results for germ cell tumors, craniopharyngioma, bladder sarcoma as well as skull base tumors [4-6,26,27].

To fully evaluate the long-term potential of particle therapy in pediatric patients requires much longer follow-up, which is the main downside in all current publications on particle therapy. Especially to completely understand the risk profile towards reduction of secondary malignancies, longer observation is required. Comparative data of protons and photons have suggested such a potential effect [28]. With respect to carbon ion radiotherapy and the distinct radiobiological properties, clinical implementation in pediatric patients should be applied cautiously due to a possible higher risk for treatment-induced secondary malignancies [29]. Therefore, this modality should be withheld for clinical situations in which the tumor is difficult to control with conventional low-LET-radiation, such as osteosarcoma. Therefore, in such a patient group the evaluation of carbon ion radiotherapy also in pediatric patients can be justified [20].

## In conclusion

The largest benefit of proton radiotherapy can be expected in the pediatric population. Therefore, establishment of particle therapy also for children and young adults was a high priority at the Heidelberg Center. Treatment of also very small children under anesthesia is possible. In general pediatric patients are treated with protons, except for specific indications within clinical protocols, e.g. osteosarcoma. Clinical protocols have been generated to evaluate the real potential of particle therapy, also with respect to carbon ions in distinct pediatric patient populations. The strong cooperation between the pediatric department and the department of radiation oncology enable an interdisciplinary treatment and stream-lined workflow and acceptance of the treatment for the patients and their parents.

## Competing interests

The authors declare that they have no competing interests.

## Authors’ contributions

SC, KH, AJ, JD, SO, CB, AEK, EM and OW provided patient care. SC, KK performed clinical analyses, assisted in data and wrote the manuscript. SE, EM AH and OJ were responsible for physical and technical conception of treatment plans and development of anesthesia equipment. SC, AJ, KH, SO and CB and JD approved treatment plans and supervised patient treatment. All authors read and approved the manuscript.

Part of this work was presented at the annual meeting of the German Society of Radiation Oncology (DEGRO), in Wiesbaden, Germany, June 2011.
